# Revealing the Efficacy of Thermostable Biosurfactant in Heavy Metal Bioremediation and Surface Treatment in Vegetables

**DOI:** 10.3389/fmicb.2020.00222

**Published:** 2020-03-10

**Authors:** Amrudha Ravindran, Arya Sajayan, Gopal Balasubramian Priyadharshini, Joseph Selvin, George Seghal Kiran

**Affiliations:** ^1^Department of Food Science and Technology, Pondicherry University, Puducherry, India; ^2^Department of Microbiology, Pondicherry University, Puducherry, India

**Keywords:** lipopeptide biosurfactant, heavy metal removal, anionic, vegetables, wastewater

## Abstract

Biosurfactants are amphiphilic molecules which showed application in the food, medical, and cosmetics industries and in bioremediation. In this study, a marine sponge-associated bacteria (MSI 54) was identified as a biosurfactant producer which showed high emulsification and surface tension-reducing property. The isolate MSI 54 was identified as *Bacillus* sp. and the biosurfactant was chemically characterized as a lipopeptide analog based on the spectral data including Fourier transform infrared spectroscopy and nuclear magnetic resonance spectroscopy. The MSI 54 lipopeptide biosurfactant was an anionic molecule which showed high affinity toward cationic heavy metals including Pb, Hg, Mn, and Cd. The heavy metal bioremediation efficacy of the biosurfactant was evaluated using atomic absorption spectroscopy, scanning electron microscopy/energy-dispersive X-ray spectroscopy, and high-resolution transmission electron microscopy analysis. When MSI 54 lipopeptide biosurfactant was added to heavy metals, this resulted in a white co-precipitate of the metal–biosurfactant complex. The heavy metal remediation efficacy of the biosurfactant at a 2.0 × critical micelle concentration (CMC) showed removal of 75.5% Hg, 97.73% Pb, 89.5% Mn, and 99.93% Cd, respectively, in 1,000 ppm of the respective metal solution. The surface treatment of farm fresh cabbage, carrot, and lettuce with 2.0 × CMC of the lipopeptide showed effective removal of the surface heavy metal contaminants.

## Introduction

Biosurfactants are amphiphilic compounds which are produced by microbial cells on their surface or secreted extracellularly. They contain hydrophobic and hydrophilic moieties that decrease the surface and interfacial tension between surfaces (Maneerat, 2005). Biosurfactants possess important characteristics that offer advantages over synthetic surfactants, which include surface and interfacial activity, tolerance to temperature, pH, and ionic force, low toxicity, availability, specificity, biocompatibility, and biodegradability (Kiran et al., 2010a; Franzetti et al., 2014). Because of their promising benefits, they are extensively used for industrial and medicinal purposes (Muthusamy et al., 2008). Heavy metals are the earth metals, and some of those are important supplements for living beings. Metals accumulate in the environment as the by-product of anthropogenic developmental activities such as tanneries, manufacturing of arms and ammunition, paint, metal pipes, batteries, mining, etc. (Singh and Cameotra, 2004), and their high accumulation can lead to serious intoxication (Mao et al., 2016). Heavy metal accumulations in the environment are persistent to degradation, but can be converted to less toxic forms (Santona et al., 2006). Biosurfactants enhance the solubility of hydrophobic compounds in the polluted environment and helps in bioremediation (Cameotra and Makkar, 2010). Among the lipopeptide biosurfactants, surfactin and its analogs are the most widely studied, and they consist of a lipid chain connected to a short linear or cyclic peptide (Peypoux et al., 1991). Our previous reports on the production of biosurfactants by marine actinobacteria showed that metal ions such as FeCl_3_, CuSO_4_, MnCl_2_, and MgSO_4_ significantly influenced biosurfactant production (Kiran et al., 2010b). A study by Cameotra and Makkar (2010) reported that the addition of metal ions increased biosurfactant production. Heavy metal remediation by a biosurfactant occurs either by association of the complex with the free-form metal residues or by accumulation at a solid solution interface, which leads to the direct interaction of the metal and the biosurfactant (Miller, 1995). By the process of desorption, the biosurfactant–metal complex leaves the soil surfaces and form micelles. The biosurfactant can be further precipitated and separated from the metals (Lee et al., 2008). According to Mulligan et al. (2001), a surfactant can be recovered from the metal complex by lowering the pH below 5. The heavy metals recovered in the bioremediation process can be reused, which reduces the need for ore mining or synthesis of heavy metals.

In this study, a biosurfactant produced by a marine sponge-associated bacteria, MSI 54, was chemically characterized as a lipopeptide. The produced MSI 54 lipopeptide was anionic and effectively remediates cationic heavy metals such as Cd^2+^, Pb^2+^, Hg^2+^, and Mn^2+^. The heavy metal remediation efficacy of the biosurfactant on the surface treatment of fresh vegetables and wastewater was evaluated using atomic absorption spectroscopy (AAS), scanning electron microscopy (SEM)/energy-dispersive X-ray spectroscopy (EDX), and high-resolution transmission electron microscopy (HRTEM) analysis.

## Materials and Methods

### Collection and Isolation of Sponge-Associated Bacteria

The marine sponge used in the study was collected from the coastal regions of Palk Bay, Rameswaram, at a depth of 2 M. The sponge samples were stored in Ziploc bags and then immediately placed in an icebox. The box was then transferred to the laboratory for the isolation of bacteria (Selvin et al., 2009). Briefly, a 1-cm^3^ portion of sponge tissue was washed with sterile water, homogenized, and then serially diluted with phosphate-buffered saline (pH 7.0). The aliquots were spread plated on nutrient agar with 2% NaCl and incubated at 27°C for 48 h. The colonies obtained were stored under −20°C.

### Screening for Biosurfactant Production

All the isolates obtained were inoculated on minimal media with 2% NaCl and incubated for a period of 5 days. After incubation, cell-free broth was obtained by centrifugation at 10,000 rpm for 15 min. The supernatant was then screened for drop collapse activity (Youssef et al., 2004), oil displacement (Gandhimathi et al., 2009), lipase (Kiran et al., 2009), hemolytic activity (Carrillo et al., 1996), emulsification index (Paraszkiewicz et al., 2002), and surface tension determination using a tensiometer.

### Identification of Biosurfactant Producer

The isolate MSI 54 was selected for further characterization studies based on the stable emulsification activity. Morphological and biochemical characteristics were studied and identified using Bergey’s *Manual of Determinative Bacteriology* and molecular identification based on 16S ribosomal DNA (rDNA) analysis. The DNA of the isolate MSI 54 was isolated and PCR amplification was performed using the 16S rDNA primer (5′-GAGTTTGATCCTGGCTCAG-3′; 5′-AGAAAGGAGGTGATCCAGCC-3′). The gene sequence obtained was blasted using NCBI MegaBLAST and phylogenetic trees were constructed in MEGA 7 version 2.0 using the neighbor joining (NJ) method.

### Optimization of Biosurfactant Production

To maximize the production of biosurfactant, a one-factor-at-a-time experiment was performed. The factors optimized include carbon, nitrogen, salt, pH, temperature, and time. The isolate MSI 54 was grown in nutrient agar media supplemented with 1% of different carbon and nitrogen sources. The carbon sources used include glucose, fructose, lactose, and starch; the organic and inorganic nitrogen sources used were beef extract, yeast extract, peptone, and ammonium chloride. Effective concentrations of the carbon (lactose) and nitrogen (peptone) sources were optimized in the range 1–5%. Production media were maintained at temperatures of 4, 28, 30, and 37°C, pH values of 2, 4, 6, 7, 8, and 10, and salt concentrations of 2–5%. All the optimization experiments were performed in triplicate.

### Extraction and Purification of the Biosurfactant

The isolate MSI 54 was inoculated into optimized production media maintained at a pH of 7.0 and incubated at 28°C on a shaker incubator (Orbitek) for 5 days. To extract the biosurfactant, the bacterial cells were separated by centrifugation and the filtrate was acidified to pH 2.0 using 2N HCl and was kept at 4°C overnight. The precipitate obtained was washed with sterile water until it reaches the pH of 7.0. The biosurfactant thus obtained was purified using column chromatography, followed by high-performance liquid chromatography (Kiran et al., 2017). Column chromatography was performed using silica gel (60–120 mesh) and elution was performed using the solvents of methanol and water (65–100, *v*/*v*) with a flow rate of 0.5 ml/min. The purity and the *R*_f_ value of the compound were checked on thin layer chromatography (TLC) with the solvent system of chloroform/methanol/water (65:25:10).

### Chemical Characterization of the Biosurfactant

The functional groups of the purified biosurfactant were determined using a Fourier transform infrared (FTIR) spectrophotometer (Bruker IFS113v FTIR spectrometer), in the range 4,000–400 cm^–1^. Gas chromatography–mass spectrometry (GC-MS) of MSI 54 was performed on PerkinElmer (GC model 680) with an oven initial temperature of 60°C for 2 min, ramp 10°C/min to 300°C, and hold 6 min. Further characterization of MSI 54 was performed using ^1^H NMR and ^13^C NMR (Bruker AVANCE III 500 MHz) and then analyzed for proton and carbon shifts.

### Determination of Ionic Characteristic of the Biosurfactant

The nature of the MSI 54 biosurfactant was analyzed using the agar double diffusion method (Van Oss, 1968). Briefly, two wells were made on 1% of soft agar and wells of the first row were filled with ionic charge. The anionic and cationic standards used were 20 mM of sodium dodecyl sulfate (SDS) and 50 mM barium chloride, and the line of precipitation was observed after incubation at 37°C for 48 h.

### Determination of the Critical Micelle Concentration (CMC)

The CMC was determined by plotting surface tension as a function of the concentration of the MSI 54 biosurfactant. It was determined as the concentration of the biosurfactant required to form micelles. The surface tension was measured using a Du Nouy ring tensiometer (Carrillo et al., 1996).

### Stability of the MSI 54 Biosurfactant

Stability of the MSI 54 biosurfactant was determined in varying temperatures, salt concentrations, and pH using column purified fraction. To evaluate stability, 0.4% (*w*/*v*) of the biosurfactant was used and the assays were performed within the ranges of pH (4.0–9.0), NaCl concentration (2–16%), and temperature (4–121°C) (Kiran et al., 2010c, 2017).

### Effect of the Biosurfactant on Heavy Metal Remediation

The heavy metals selected in this study was mercury, lead, manganese, and cadmium. For metal remediation assay, respective metal solutions of 1 g/L were prepared and treated with 0.5 × CMC, 1 × CMC, and 2 × CMC of the MSI 54 lipopeptide and incubated overnight at 37°C. The metal–biosurfactant co-precipitate obtained was separated by centrifugation at 10,000 rpm for 30 min. The residual metal contents in the treated and untreated (control) solutions were analyzed using AAS. The effective concentration of 2 × CMC of the biosurfactant and the heavy metals Pb and Cd were chosen for SEM-EDX. Quantitative elemental analysis of heavy metals in the biosurfactant–heavy metal co-precipitate was determined using SEM-EDX. To further confirm the precipitation of heavy metals, HR-TEM was performed (Tecnai G2-F30STwin, FEI, United States).

### Analysis of Heavy Metal Contamination in Vegetables

Vegetables such as cabbage, carrot, and lettuce were selected to study the effect of biosurfactant on heavy metal remediation. The vegetables were procured from the local market in Pondicherry, washed under tap water, and then cut into pieces. The vegetable pieces were soaked for 20 min in the respective wash solutions containing 2.0 × CMC of the MSI 54 lipopeptide in 1 L of distilled water and served as the test; distilled water without lipopeptide served as the control. The treated vegetables were blended in a commercial blender to yield a homogeneous paste, followed by drying completely at 80°C in a hot air oven. Dried samples of 1 g were transferred into a 50-ml glass beaker, to which 10 ml of concentrated HNO_3_ was added and incubated overnight, followed by heating on a hot plate to facilitate dryness (Nwajei, 2009). Then, 5 ml of perchloric acid was added and the supernatant was collected and diluted to 50 ml with distilled water and then subjected to AAS for the analysis of heavy metals in the vegetables.

### Heavy Metal Analysis in Wastewater

Wastewater was collected from an industry in Pondicherry. To 1 L of water, 2 × CMC of the biosurfactant was added with continuous stirring. After 48 h of treatment, the formed biosurfactant–metal co-precipitate was separated by centrifugation and the precipitate thus obtained was collected by centrifugation at 10,000 rpm for 10 min. The residual metal contents in the treated and untreated (control) samples were analyzed using UV/Vis and AAS.

## Results

### Isolation and Screening of Biosurfactant-Producing Sponge-Associated Bacteria

A total of 57 discrete bacterial colonies were isolated from the marine sponge *Agelas clathrodes*. Among the isolates, nine isolates showed positive on the drop collapse test and oil displacement activity. Out of which, the isolate MSI 54 showed the highest emulsification index of 60% and surface tension reduction of 30 mN/m; thus, MSI 54 was selected for further characterization studies.

### Identification of Biosurfactant Producer

**FIGURE 1:**
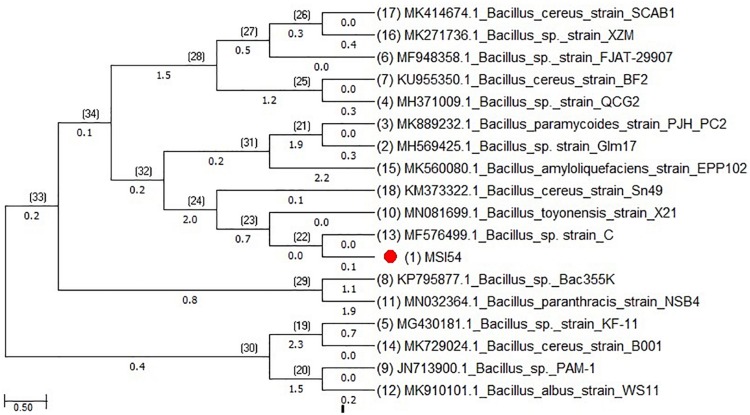
Phylogenetic tree of MSI 54 constructed using the neighbor joining (NJ) method showing representatives of *Bacillus* sp.

The morphological and phenotypic identification of the isolate MSI 54 was performed and was identified as Gram-positive cocci. The 16S rDNA sequence showed closest matches with *Bacillus cereus* and strains, and therefore, the isolate MSI 54 was taxonomically identified as *Bacillus* sp. (Figure 1). The sequence thus obtained was deposited in GenBank with the accession number MN342149.

### Optimization of Biosurfactant Production

**FIGURE 2:**
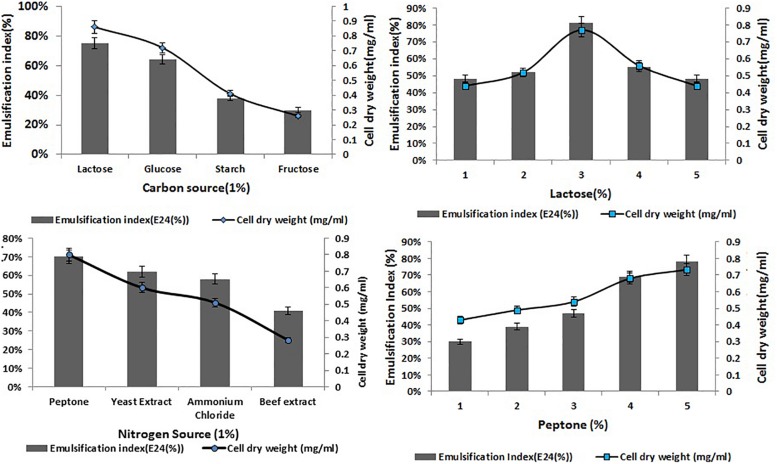
Optimization of various carbon and nitrogen sources and the effect of lactose and peptone on the cell dry weight of MSI 54 and emulsification index.

**FIGURE 3:**
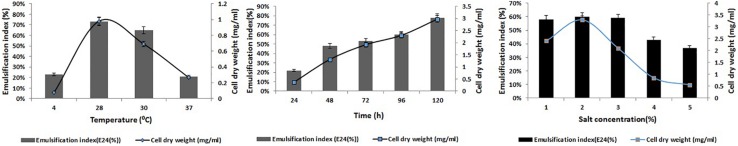
Optimization of temperature, time, and salt concentrations for the production of the MSI 54 biosurfactant.

Optimization of the factors influencing the growth of MSI 54 was performed by using the one-factor-at-a-time procedure. The biosurfactant production and the emulsification index were higher in 1% lactose, followed by glucose, fructose, and starch at the same concentration. Peptone was found to be the most effective nitrogen source, followed by yeast extract, beef extract, and ammonium chloride (Figure 2). The optimum yield of biosurfactant was achieved at 5% of peptone and 3% lactose. Among the salt concentrations used, 2% NaCl, a temperature of 28°C, and an incubation period of 5 days yielded an optimum emulsification activity of 81% (Figure 3).

### Structural Characterization of the Biosurfactant

**FIGURE 4:**
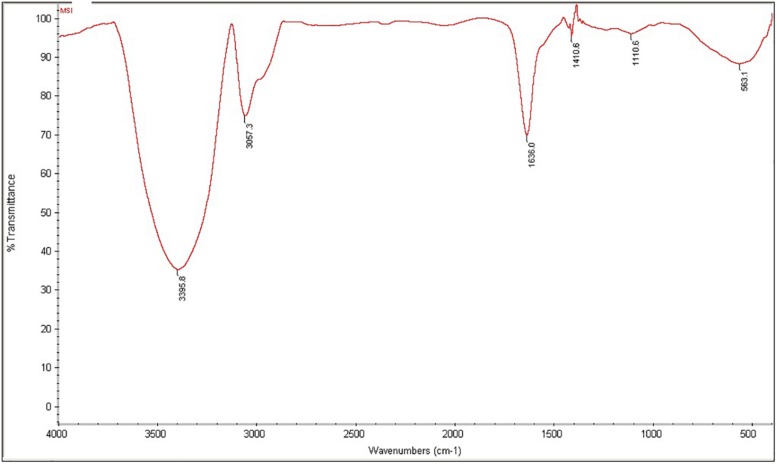
Fourier transform infrared (FTIR) spectra of the column-purified active fraction of the biosurfactant produced by MSI 54.

The purified MSI 54 biosurfactant showed a single spot in TLC with an *R*_f_ value of 0.86. The infrared spectrum showed a characteristic broad absorption peak at 3,395.8 cm^–1^, which indicates the presence of a N–H stretching mode, and a peak at 3,057.3 cm^–1^, which may be due to the presence of unsaturated alkenes and/or aromatic rings. The characteristic peak at 1,636 cm^–1^, attributed to stretching of the CO–N bond vibrations, may be due to the stretching in the peptide bond of the lipopeptide molecule (Figure 4). The peak at 1,410.6 cm^–1^ may be due to the presence of a methyl group (CH_3_), and 1,110.6 cm^–1^ indicates the C–O–C stretching of ethers. The fatty acid moiety of MSI 54 was predicted through a GC-MS analysis as a palmitic acid vinyl ester (C_18_H_34_O_2_) with a mass of 282.46 (Supplementary Figure S1).

### Nuclear Magnetic Resonance of MSI 54

**FIGURE 5:**
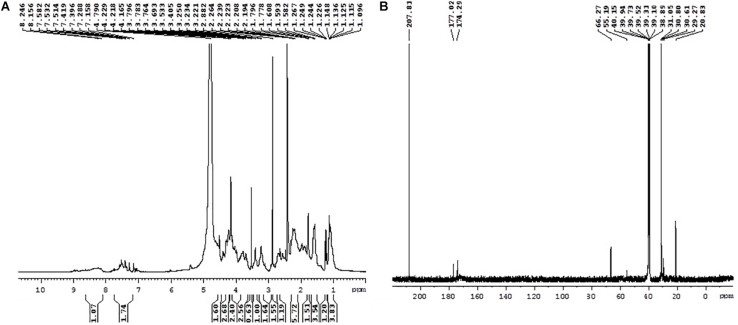
^1^H NMR **(A)** and ^13^C NMR **(B)** spectra of MSI 54.

The ^1^H NMR spectra showed that the peaks in the range 2.24–1.5 ppm represent the presence of aliphatic -CH_2_ protons. The peaks at 4.791 and 4.165 ppm represent the CH_2_OH group of amino acids. Several multiplicity peaks were present in the range 6.01–8.97 ppm, which show the presence of aromatic amide protons. The signals in the range 2.19–2.26 ppm show the presence of alpha carbon protons attached to the carbonyl group (Figure 5A). The presence of carbonyl groups at 2.19–2.26 ppm and the amide group in the range 7.397–7.778 ppm confirms the conjugation of the amino and carboxyl groups. The peak at 7.397 ppm confirms the presence of an aromatic ring.

^13^C NMR also confirms the presence of the functional groups obtained in ^1^H NMR. The peaks ranging between 20.83–30.80 and 20.83–55.19 ppm confirm the presence of primary, secondary, tertiary, and quaternary alkyl groups. The peaks at 40.15 and 55.19 ppm reveal the presence of alpha carbon protons attached to oxygen molecules (Figure 5B). The peak at 66.27 ppm represents the carbon atoms attached to hydroxyls or amines, the peaks at 174.29 and 177.02 ppm show the presence of carboxylic acid, and the peaks at 207.81 ppm indicate the presence of an aldehyde or a ketone group.

### Nature of the Lipopeptide and Critical Micelle Concentration of MSI 54

Agar double diffusion assay showed the formation of precipitation lanes between the MSI 54 lipopeptide and the cationic barium chloride, while no precipitation lane was formed between the MSI 54 lipopeptide and the anionic SDS, which demonstrates the anionic nature of the biosurfactant. The CMC of the MSI 54 biosurfactant was 10 mg/L. At this concentration, the surface tension of water dropped from 69.0 to 30.0 mN/m, beyond which the surface tension remained the same.

### Stability Studies

**FIGURE 6:**
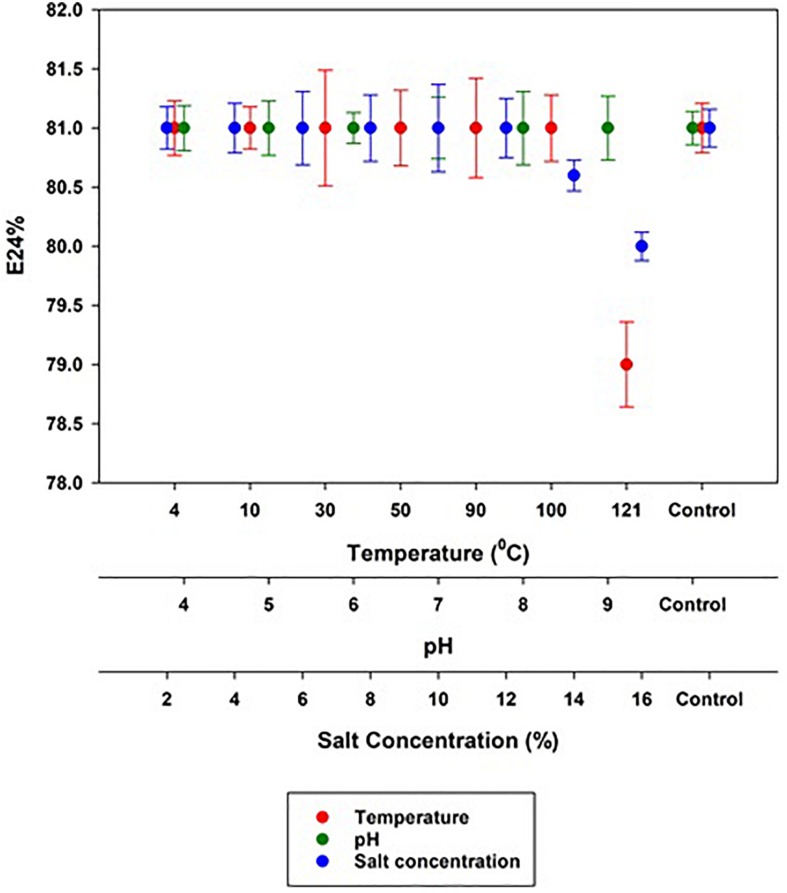
Stability of the MSI 54 lipopeptide at varying temperature, pH, and salt concentrations.

The stability of the MSI 54 biosurfactant determined in varying salt concentrations, pH, and temperatures showed an emulsification activity similar to that of the control. The emulsification index (81%) was retained even at higher and lower pH levels of 4.0–9.0 and salt concentrations of 2–12%. The MSI 54 lipopeptide biosurfactant was found to be thermostable in all the ranges of temperature tested, from 4 to 121°C, with a slight change in emulsification of 79% achieved at 121°C (Figure 6).

### The Effect of Biosurfactant on Heavy Metal Remediation

Biosurfactant-mediated heavy metal remediation was observed by AAS. The results obtained showed a reduction in the heavy metals when treated with various concentrations of the MSI 54 biosurfactant. The heavy metals, when treated with the MSI 54 biosurfactant, resulted in a white co-precipitate of the metal–biosurfactant complex. The percentage remediation of various metals varied with the concentration of the MSI 54 biosurfactant. The results obtained in AAS showed that a 0.5 × CMC of MSI 54 aided in the removal of 15% Hg, 42.2% Pb, 22.3% Mn, and 48.7% Cd. When the concentration of the biosurfactant was increased to 2.0 × CMC, higher remediation efficiencies were obtained—75.5% Hg, 97.73% Pb, 89.5% Mn, and 99.93% Cd—in the 1,000-ppm of respective metal solution. The obtained AAS results revealed that 2.0 × CMC of the MSI 54 lipopeptide remediated nearly 100% of Pb and Cd, followed by Mn and Hg (Supplementary Figure S2). The results showed that the addition of a higher concentration of the biosurfactant to the metal solution reduces the surface tension and, subsequently, the biosurfactant aggregate with the metal solutions and forms a precipitate.

### SEM-EDX and TEM Analysis

**FIGURE 7:**
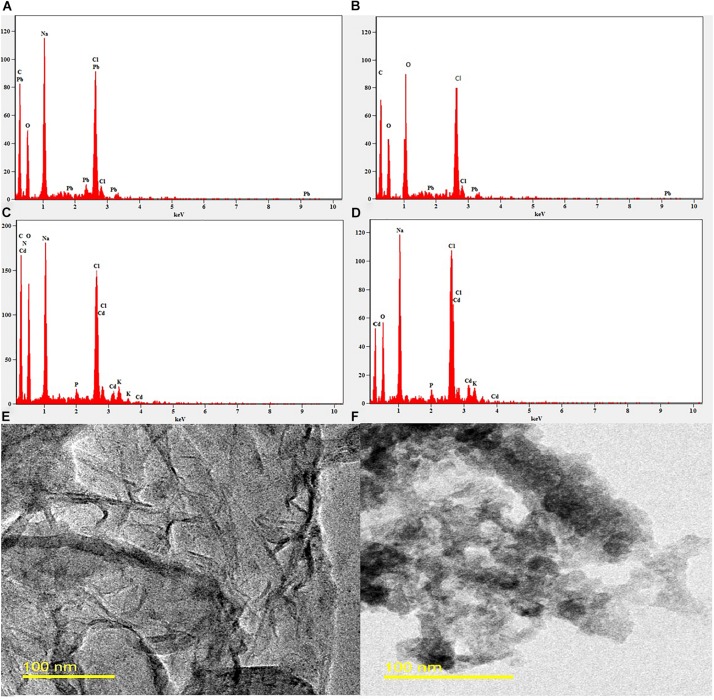
EDX and TEM image analysis. **(A)** EDX of the control Pb solution. **(B)** EDX of the Pb solution treated with MSI 54 biosurfactant. **(C)** EDX of the control Cd. **(D)** EDX of the Cd solution treated with MSI 54 biosurfactant. **(E)** TEM image of the MSI 54 biosurfactant. **(F)** A representative image of Cd solution treated with biosurfactant.

The 2.0 × CMC of the MSI 54 lipopeptide biosurfactant was selected for the analysis of heavy metals such as Pb and Cd in SEM-EDX analysis. When heavy metals were treated with the biosurfactant, it forms a complex association of metals and the biosurfactant by the process of electrostatic interaction. The elemental composition of heavy metals and the remediation rate were analyzed using EDX spectra. EDX analysis of the MSI 54 biosurfactant and heavy metal solutions treated with the biosurfactant was shown in Figure 7. The SEM image analysis showed that the heavy metals were reduced upon treatment with the biosurfactant. The TEM analysis showed efficient entrapment of the heavy metals with the biosurfactant. The entrapped metals were observed as electron-dense dark spots surrounded by the biosurfactant.

### Effect of the MSI 54 Lipopeptide on Heavy Metal Removal From Vegetable Surfaces

**FIGURE 8:**
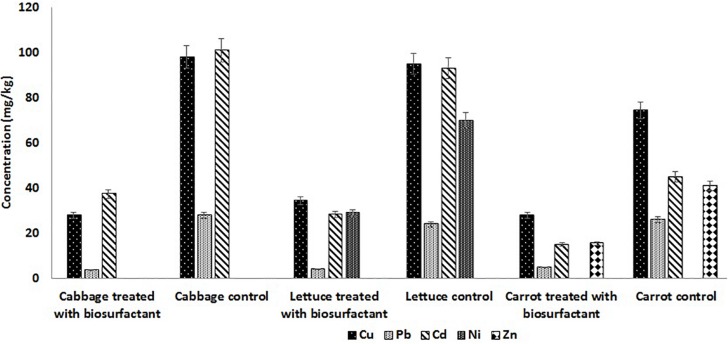
MSI 54 lipopeptide in surface treatment of vegetables showed heavy metal remediation in the vegetables treated with MSI 54.

The surface treatment of fresh cabbage, carrot, and lettuce was performed using 2.0 × CMC of the lipopeptide and the leachate was analyzed on AAS. The results obtained showed that surface treatment of cabbage, carrot, and lettuce with the MSI 54 lipopeptide effectively remediated the surface contaminants, the heavy metals. Atomic absorption analysis of the nitric acid-digested samples of vegetables (control) showed the presence of heavy metals such as copper, lead, and cadmium (Figure 8). The results of the cabbage samples treated with the biosurfactant solution showed that the concentrations of copper reduced by 71.4%, lead 86.79%, and cadmium 62.93%. Similarly, carrots treated with the biosurfactant solution showed a reduction in copper by 63.74%, lead 83.4%, zinc 62.2%, and cadmium 69.5%; in lettuce, there were reductions of copper 62.4%, lead 80.77%, cadmium 66.77%, and nickel 59%. The study revealed that the vegetables were heavily contaminated with heavy metals, which were absorbed on the plant surface from the soil, and water and biosurfactant could be used as a wash solution to remove the heavy metal contaminants from the surface of vegetables.

### Biosurfactant MSI 54 in Wastewater Heavy Metal Remediation

**FIGURE 9:**
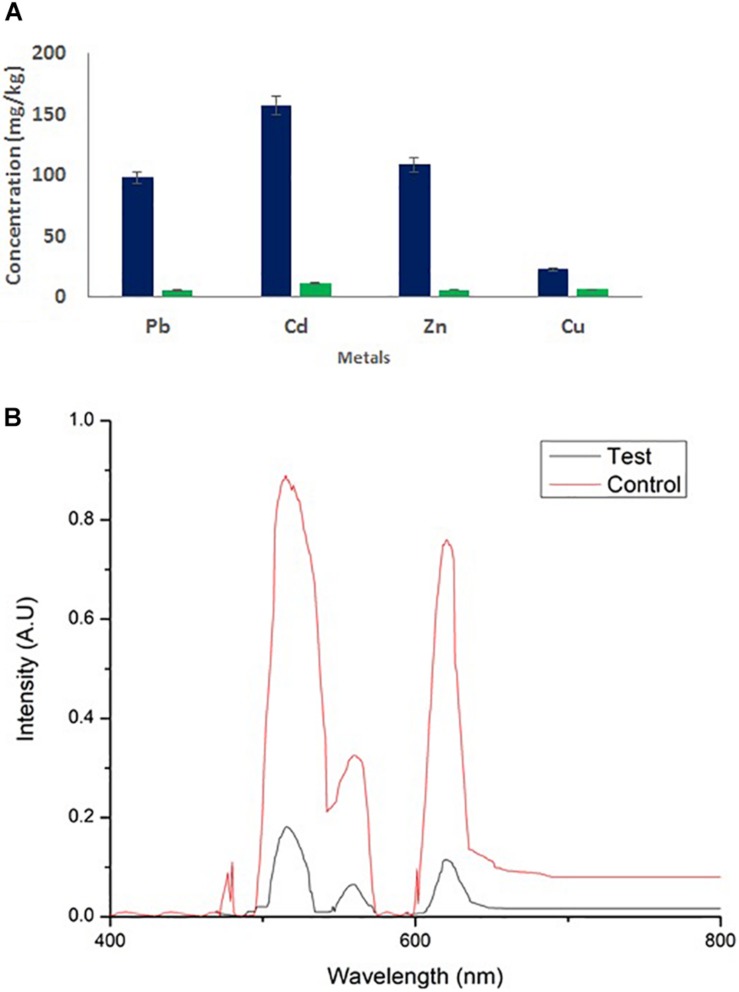
**(A)** Atomic absorption spectroscopy and **(B)** UV/Vis analysis of wastewater and its heavy metal remediation using MSI 54 lipopeptide.

Wastewater treated with 2 × CMC of the MSI 54 lipopeptide biosurfactant showed a reduction in heavy metal concentrations. Atomic absorption spectroscopy showed the remediation of heavy metals in the biosurfactant-treated wastewater. Similar results were obtained in UV spectroscopy, with absorption peaks at 515 nm for Pb and Cd, 560 nm for Cu, and 620 nm for Zn, with reduction in the metals Cu (76.07%), Zn (95.1%), Pb (95.28%), and Cd (93.13%) when compared to the untreated water (Figure 9).

## Discussion

Marine microorganisms are well known for the production of bioactive compounds, and the biosurfactants produced from those are known for biofilm disruption, antimicrobial compounds, hydrocarbon degradation, and as emulsifiers in the food industry (Kiran et al., 2010a, b, 2017). In this study, a marine sponge-associated bacteria, MSI 54, was selected for biosurfactant production based on the stable emulsification activity. The 16S rDNA sequence of MSI 54 was analyzed using the MegaBLAST tool and was identified as a *Bacillus* sp. Numerous studies reported lipopeptide production from marine *Bacillus* sp. (Dhasayan et al., 2015; Chen et al., 2017; Rozas et al., 2017; Wang et al., 2019; Wu et al., 2019). The chemical characterization of MSI 54 using FTIR and NMR showed that the biosurfactant was a lipopeptide analog (Peypoux et al., 1991; Chen and Juang, 2008; Kiran et al., 2017; da Rocha et al., 2018). The anionic characteristics of the lipopeptide MSI 54 were confirmed using the agar double diffusion technique. The lipopeptides from *Bacillus* species are known for their anionic nature (Varadavenkatesan and Murty, 2013). The CMC of the MSI 54 biosurfactant was found to be 10 mg/L, which reduced the surface tension from 69.0 to 30.0 mN/m, and the CMC of the lipopeptide from *Bacillus subtilis* K1 was found to be 17.5 μg/ml (Pathak and Keharia, 2014). Stability studies showed that the activity of the MSI 54 biosurfactant was not affected at higher temperature, pH, and salt concentrations, suggesting its environmental applications such as heavy metal remediation and metal recovery from the complex using ultrafiltration (Ferella et al., 2007). Several lipopeptide biosurfactants from marine sponge-associated bacteria have been reported (Gandhimathi et al., 2009; Kiran et al., 2010b, 2017; Sharafi et al., 2014). However, the MSI 54 lipopeptide biosurfactant seems to be unique based on GC-MS analysis; its stability and emulsification index were found to be higher than those reported. The GC-MS analysis showed the fatty acid moiety as a palmitic acid vinyl ester (C_18_H_34_O_2_) with a mass of 282.46. The *R*_f_ value of MSI 54 was found to be 0.86, which was different from the *R*_f_ value of surfactin reported as 0.72 (Yakimov et al., 1995), 0.35–0.45 (Zeriouh et al., 2011), and 0.5–0.55 (Kowall et al., 1998). Palmitic acid vinyl esters are known for the production of sucrose monoesters which are used in the food industry as emulsifiers (Yoko and Inge, 2011). The emulsification index reported for surfactin produced by *B. subtilis* were 68% with kerosene (Haddad et al., 2009), 69% with crude oil (Nitschke and Pastore, 2006), 59.1% with kerosene (de Faria et al., 2011), and 55% with hexadecane (Gudiña et al., 2015). For *Bacillus brevis*, this was 71.89 ± 0.56% with crude oil (Mouafi et al., 2016). The stability of MSI 54 was higher than that reported (Gandhimathi et al., 2009; Sharafi et al., 2014; Jha et al., 2016). The MSI 54 lipopeptide effectively remediated heavy metals such as mercury, lead, cadmium, and manganese at 2.0 × CMC. The concentration of the biosurfactant used in this study for heavy metal removal was lower when compared to the 10 × CMC reported (Das et al., 2008). Experiments were performed by Mulligan et al. (2001) for the remediation of heavy metals using surfactin, rhamnolipid, and sophorolipid and observed that surfactin remediated 15% of copper and 6% of zinc in metal-contaminated soil. According to Açıkel (2011), anionic surfactants showed more affinity to form complex with metal ions by the process of surface sorption or precipitation of the complexes. TEM image analysis showed the heavy metal biosurfactant association as an electron-dense metal visualized as dark spots engulfed by the biosurfactant. The chemical groups and a few heteroatoms such as amine/carbonyl and hydroxyl present in the functional groups of biosurfactant participate in the formation of a metal complex (Ławniczak et al., 2013). The data obtained by AAS were further confirmed on SEM-EDX for the representative metals such as Pb and Cd, and the result obtained showed that the MSI 54 lipopeptide was highly effective in the bioremediation of these heavy metals. Zhu et al. (2013) demonstrated the use of a lipopeptide biosurfactant to improve the adsorption and desorption capability for various metal ions of Na-MMT clay. The MSI 54 biosurfactant effectively remediated the Cu (76.07%), Zn (95.1%), Pb (95.28%), and Cd (93.13%) present in wastewater. The use of the MSI 54 lipopeptide as a surface treatment of farm-fresh cabbage, carrot, and lettuce showed effective removal of the surface-bound heavy metals. The permissible limits of copper, lead, and cadmium recommended by the WHO/FAO in vegetables and fruits are 40, 0.3, and 0.2 mg/kg (Husain et al., 1995). The study revealed that the amounts of heavy metals found in vegetables such as carrot, lettuce, and cabbage were much more than the permissible limits determined by the WHO/FAO. The accumulation of heavy metals in the vegetables indicates heavy metal contamination. They were directly incorporated into the human food chain and cause serious health problems, such as kidney and gastrointestinal failure, depression, osteoporosis, cardiovascular problems, hematous, tubular and glomerular dysfunction, cancers, etc. (Agency for Toxic Substances and Diseases Registry [ASTDR], 2005; World Health Organization [WHO], 2009). This study suggests the use of biosurfactant as a surface treatment solution for vegetables as well as for the removal of heavy metals in polluted areas.

## Conclusion

In this study, MSI 54 *Bacillus* sp. was isolated from a marine sponge (*A. clathrodes*). The anionic lipopeptide biosurfactant produced by this *Bacillus* strain showed high affinity against cationic heavy metals such as Pb, Cd, Hg, and Mn. Based on the stable emulsification activity of this biosurfactant at high temperature, pH, and salt concentrations, we explored possible environmental applications for the remediation of heavy metal contamination. The EDX and AAS analyses revealed the effective removal of Pb and Cd by the MSI 54 lipopeptide. The study concludes that this biosurfactant could be used as a surface treatment solution for the removal of heavy metals from vegetables as well as for the remediation of heavy metal contamination of different environments.

## Data Availability Statement

All datasets generated for this study are included in the article/Supplementary Material.

## Author Contributions

AR performed the experiment. AS and GP helped in sponge collection and screening assays. GK wrote the manuscript. JS designed the work.

## Conflict of Interest

The authors declare that the research was conducted in the absence of any commercial or financial relationships that could be construed as a potential conflict of interest.
