# Supplementary material for: Revealing the Efficacy of Thermostable Biosurfactant in Heavy Metal Bioremediation and Surface Treatment in Vegetables
Source: Front Microbiol. 2020 Mar 10;11:222. doi: 10.3389/fmicb.2020.00222 (PMC7076089; doi:10.3389/fmicb.2020.00222)
Supplement: Supplementary file 1 [file Data_Sheet_1.pdf]

# Revealing the efficacy of thermostable biosurfactant in heavy metal bioremediation and surface treatment in vegetables

Amrudha Ravindran[1], Arya Sajayyan[1], Gopal Balasubramaniam Priyadharshini[2], Joseph Selvin[2], G. Seghal Kiran[1*]

[1]Department of Food Science and Technology, Pondicherry University, India

[2]Department of Microbiology, Pondicherry University, India

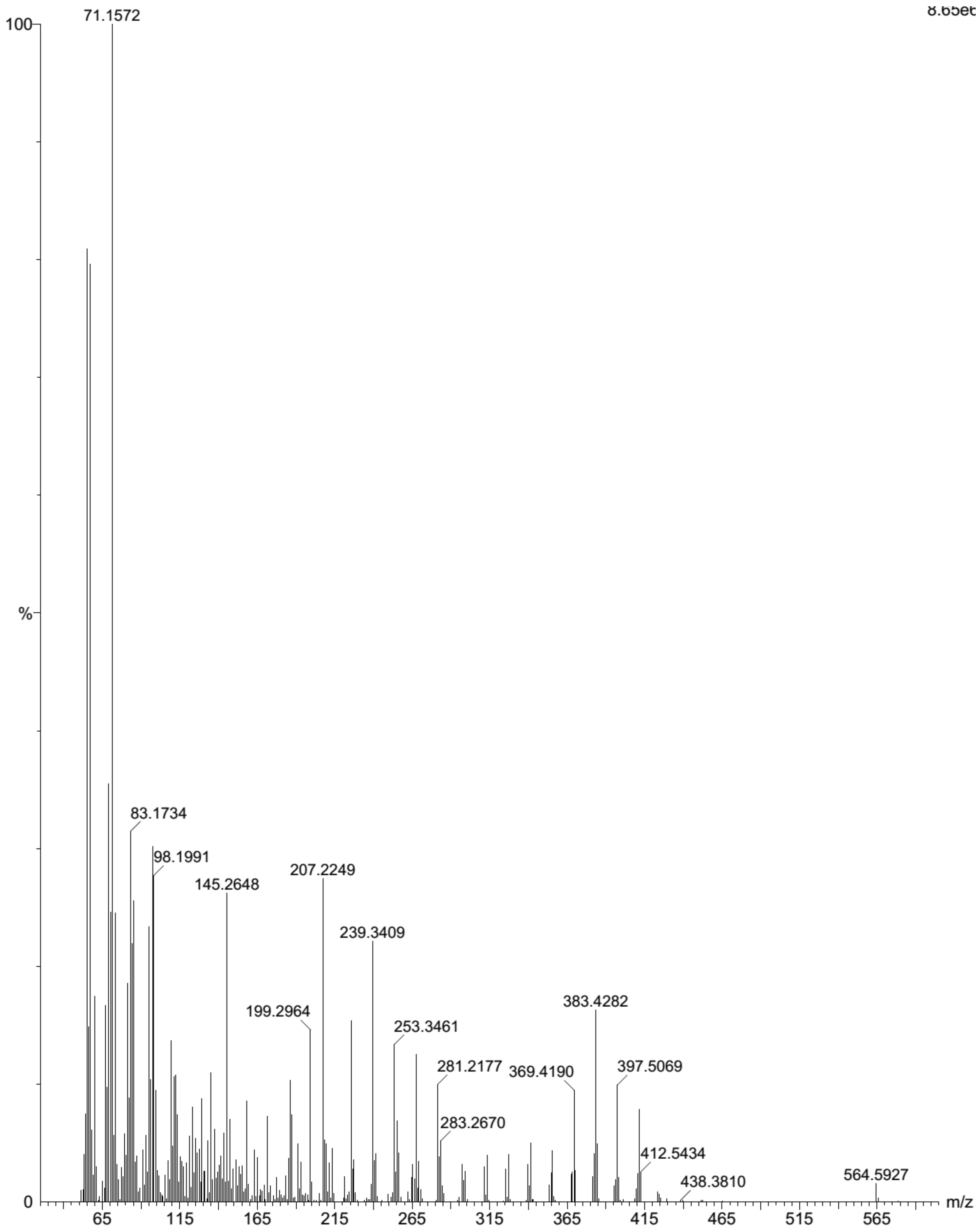


**Suppl. Fig. S1** Mass spectrum of MSI 54, showed fatty acid moiety as palmitic acid vinyl ester ($C_{18}H_{34}O_2$).

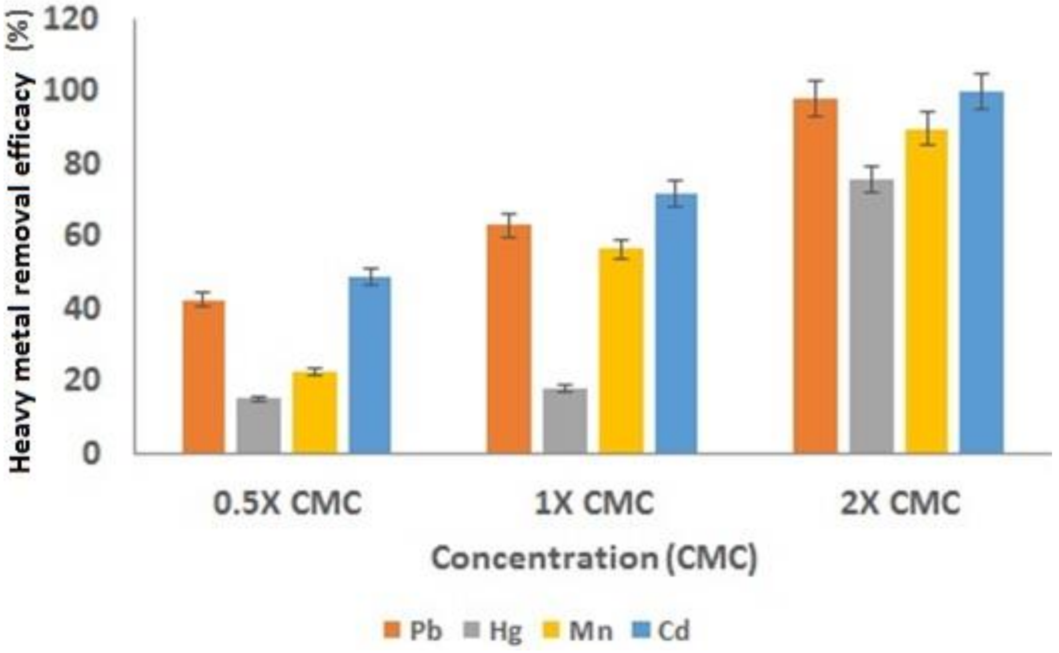


**Suppl. Fig S2)** Heavy metal remediation efficacy of biosurfactant at varying concentrations 0.5 x CMC, 1 .0 x CMC and 2.0 x CMC.
